# Correction to “Diversity and Relative Abundance of Avian Species in the Wetland Area Northwest of Lake Abaya, Southern Ethiopia”

**DOI:** 10.1155/tswj/9817625

**Published:** 2025-10-10

S. Bekele and W. Tekalign, “Diversity and Relative Abundance of Avian Species in the Wetland Area Northwest of Lake Abaya, Southern Ethiopia,” *The Scientific World Journal* 2023 (2023): 9964189, https://doi.org/10.1155/2023/9964189

In the article, References 44 and 45 were erroneously retained during the production process and should have been omitted.

We apologize for this error.

